# Supplementary material for: Alternative sulphur metabolism in the fungal pathogen Candida parapsilosis
Source: Nat Commun. 2024 Oct 24;15:9190. doi: 10.1038/s41467-024-53442-8 (PMC11502921; doi:10.1038/s41467-024-53442-8)
Supplement: Supplementary file 1 — Supplementary Information [file 41467_2024_53442_MOESM1_ESM.pdf]

# Alternative sulphur metabolism in the fungal pathogen *Candida parapsilosis*

Lisa Lombardi[1]*, Letal I. Salzberg[2], Eoin Ó Cinnéide[1], Caoimhe O'Brien[1], Florent Morio[3], Siobhan A. Turner[1], Kevin P. Byrne[2], Geraldine Butler[1]*

[1]School of Biomolecular and Biomedical Science, Conway Institute, University College Dublin, Belfield, Dublin, Ireland; [2]School of Medicine, Conway Institute, University College Dublin, Belfield, Dublin, Ireland; [3]Nantes Université, CHU Nantes, Cibles et Médicaments des Infections et de l'Immunité, UR1155, Nantes, France.

*Corresponding authors. Emails: lisa.lombardi@ucd.ie; gbutler@ucd.ie

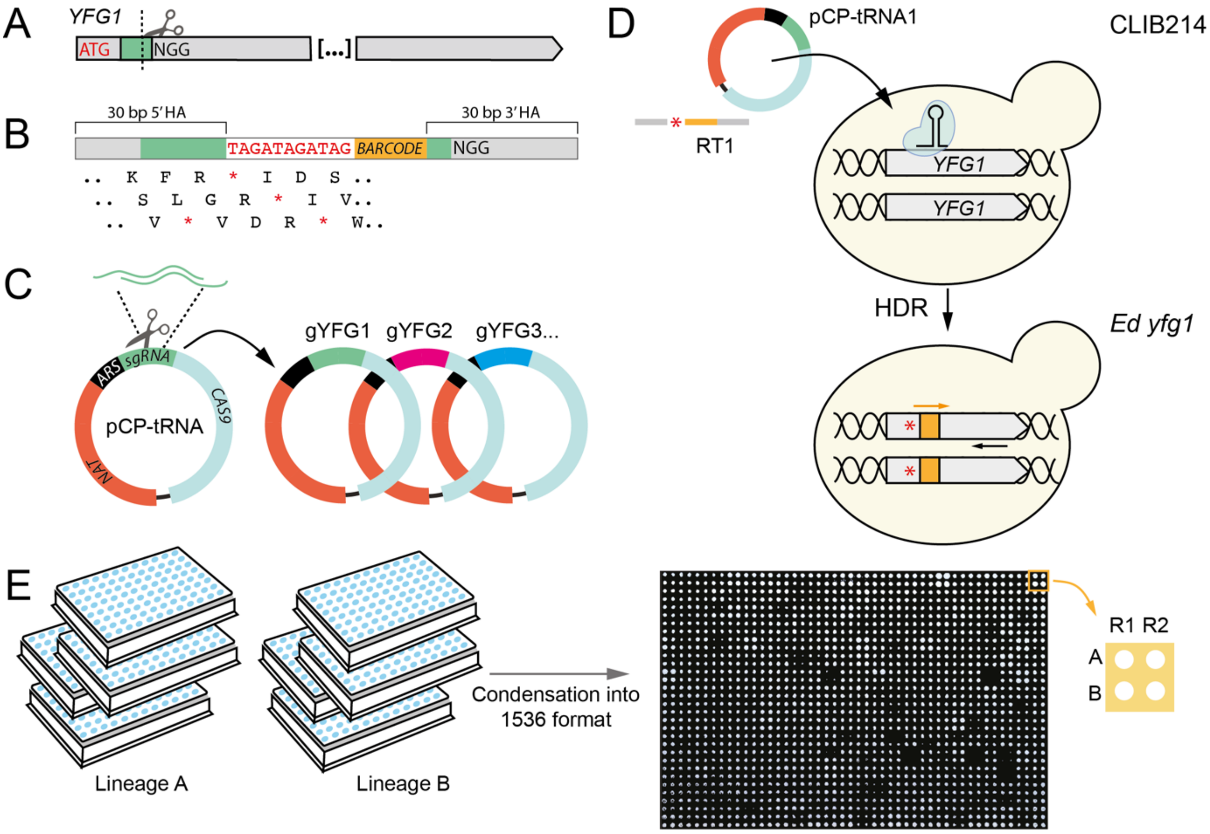


**Supplementary Figure 1. Features of the mutant collection in *C. parapsilosis*.** The figure depicts the workflow for the generation of the CRISPR-Cas9 edited mutants and the features of the complete collection, including the deletion strains generated in[1]. **(A)** For the generation of CRISPR-Cas9 edited strains, guides were designed to drive Cas9 cleavage at the beginning of the coding region of each desired gene (*YFG*, Your Favourite Gene) (e.g. *YFG1*). Details on guide design are provided in the Methods section. **(B)** The Repair Templates (RTs) were designed to introduce a premature stop codon and a unique barcode following Homology Directed Repair (HDR) with the target locus on the genome. HAs = Homology Arms (HAs). **(C)** A library of plasmids targeting the genes of interest was generated by cloning the short dsDNA guides (e.g. *gYFG1*, *gYFG2*) into the pCP-tRNA empty plasmid, which harbours *CAS9*, the Autonomously Replicating Sequence, and a nourseothricin marker cassette (*NAT*) [2]. **(D)** The simultaneous transformation of the pCP-tRNA (e.g. pCP-tRNA1) targeting a gene (e.g. *YFG1*) and the corresponding RT (e.g. RT1) resulted in the insertion of a premature stop codon (red asterisk) in both alleles by HDR, thus generating an edited strain (e.g. Ed yfg1). A primer annealing on the barcode was used to confirm the modification in PCR. **(E)** Two independent lineages (A and B) were obtained for most of the strains. The mutant collection was condensed using a Singer Instruments ROTOR HDA in a 1536 format onto a YPD plate (source plate). Each mutation was represented in two technical replicates (R1 and R2) and two biological replicates (A and B). The source plate was then replicated onto the phenotyping plates.

# Supplementary Note 1

**Phenotypic effects of disruptions: baseline growth in YPD, SD + AS, SC + AAs**

The phenotypic screen used three different base media: i) YPD media supplemented with different chemical stressors; ii) Synthetic Defined (SD) media supplemented with ammonium sulphate (AS) or alternative nitrogen sources; and iii) Synthetic Complete (SC) media supplemented with all amino acids (AAs) or lacking specific amino acid classes/adenine/histidine.

We first measured the baseline growth of the *C. parapsilosis* disruptions by assessing the colony size on solid media. We tested the growth of the mutant strains on i) YPD media (without any chemical stressors); ii) SD media supplemented with AS, the preferred nitrogen source, and iii) SC media supplemented with all the AAs (Supplementary Data 2) in comparison to relevant control strains, *C. parapsilosis* CLIB214 and *C. parapsilosis* CPRI[1]. Because the colony sizes of *C. parapsilosis* CLIB214 and CPRI were similar within each of the three conditions tested, we defined $\text{Growth}_{(\text{CTRL})}$ as the mean of the normalized observations for both strains, and the Growth Ratio for each mutant strain in the library as (normalized size of mutant colony/Growth(CTRL)) (Supplementary Data 2 and Methods). For most strains the Growth Ratio was close to 1, meaning that the mutation introduced did not result in a baseline growth defect (Supplementary Figure 2). However, 21 strains showed a growth defect (Growth Ratio <= 0.5) in at least one media (Supplementary Figure 2).

Mutant strains disrupted in *SFP1* (*CPAR2_807380*), *GRR1* (*CPAR2_100650*), *RSA1* (*CPAR2_102070*) and *FIP1* (*CPAR2_100690*) showed a severe growth defect on the three media tested (Growth Ratio 0-0.2, Supplementary Figure 2). Loss of function of the master transcriptional regulator Sfp1 and the component of the SCF ubiquitin-ligase complex Ggr1 required for cell cycle progression are associated with a dramatic decrease in cell size in *C. albicans*[3,4]. In *Saccharomyces cerevisiae* loss of function of Rsa1 is associated with decreased cell size and abnormal cell cycle progression[5].

Deleting the transcriptional repressor *TUP1* (*CPAR2_109520*) also affected growth (Growth Rate 0.3-0.4), and a similar effect was observed for the *Ed ssk2* (*CPAR2_403060*), *Ed sak1* (*CPAR2_401830*), and *Ed pho23* (*CPAR2_407350*)

mutants (Supplementary Figure 2). In *C. albicans*, mutating the Ssk2 kinase determines defects in colony morphology, *in vivo* proliferation[4], and growth on different media[6], while deleting *SAK1* does not result in reduced growth, but it has effects on colony morphology[7]. In *S. cerevisiae*, deletion of *PHO23* results in decreased vegetative growth rate[8].

Disruption of *SAH1* (*CPAR2_101020*), responsible for the conversion of S-adenosyl homocysteine to homocysteine in the methyl cycle, only marginally affected growth on YPD, but its effect was more evident on YNB and SC (Growth Rate < 0.2). However, *SAH1* is essential in both *C. albicans* and *S. cerevisiae*[9,10], raising the possibility that a truncated version of the protein, partially maintaining gene function, may be produced in the *C. parapsilosis* edited strain.

Targeting Vacuolar Protein Sorting (VPS) proteins 15 and 34 (encoded by *CPAR2_602810* and *CPAR2_206880*, respectively) also resulted in a growth defect. In *C. albicans*, loss of Vps34 function results in a plethora of phenotypes, including a decreased rate of vegetative growth[11-13]. Our library included two independent *C. parapsilosis* Vps34 mutants: a deletion mutant generated by Holland et al. [1], and a CRISPR-Cas9 edited strain. Notably, the phenotype was consistent in both. A decreased growth rate was also observed for the *Ed swi4* and *Δswi4* mutants (*CPAR2_109060*), similarly to what reported for *C. albicans*[4].

Several mutations affected growth specifically on only one of the media. Deleting the GATA transcriptional activator *GLN3* (*CPAR2_101010*), required for utilization of nonpreferred nitrogen sources in *C. parapsilosis,* abolished the ability to grow on SD (YNB with ammonium sulfate)[14] (Supplementary Figure 2). Similarly, deleting *GCN4* (*CPAR2_806570*), encoding the major activator of the General Amino Acid Control (GAAC) supra-pathway in response to starvation for any amino acid, resulted in a growth defect on SD and SC media[14]. Loss of function of *ACE2* (*CPAR2_204370*), *NRG1* (*CPAR2_300790*), and *BNA2* (*CPAR2_100040*) specifically affected growth on SC in our experiment.

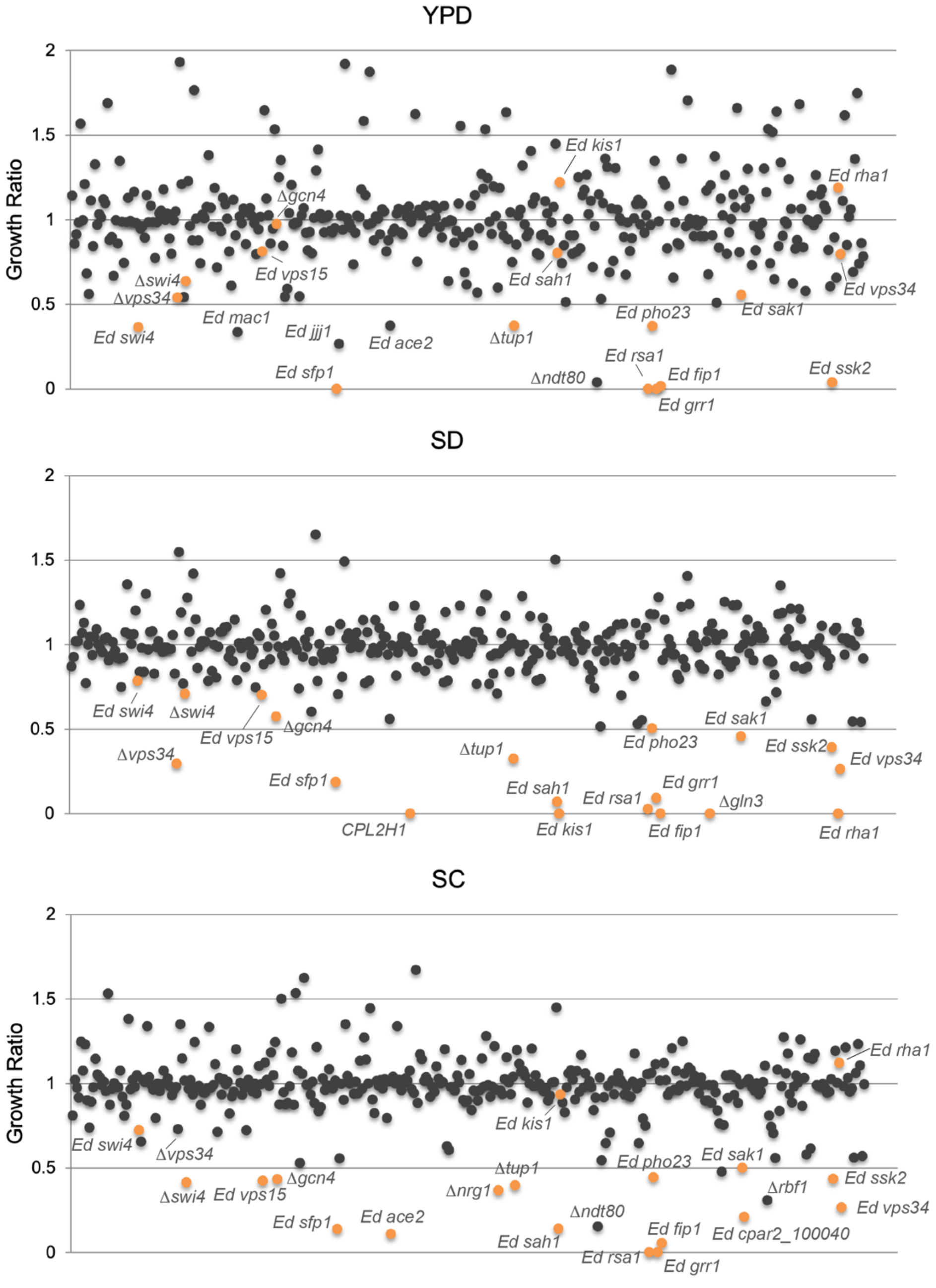


**Supplementary Figure 2. Growth of the mutant library on solid control media.** The panels depict the distribution of the Growth Rate values of the mutant strains when grown at 30°C on YPD, SD, and SC media for 24h (YPD) of 48h (SD, SC). For each strain, the Growth Ratio was calculated as (normalized size of colony/Growth$_{(CTRL)}$, where Growth$_{(CTRL)}$ = mean of normalized growth of CLIB214 and CPRI) (see Methods). The genes are labelled based on their characterized orthologs in *Candida albicans*. Mutants that showed a Growth Ratio in the 0-0.5 range in at least one condition are highlighted in orange. Strains that met this criterion but showed either different behaviour between the two lineages (*Ed ace2* and *Ed jjj1* on YPD; *Δndt80* on YPD and SC; *Δvps34* on SC) or pinning inaccuracy (*Δrbf1* on SC) in specific conditions are indicated with the name label but coloured in grey. *Ed mac1* showed a growth defect only on YPD (Growth Rate 0.34); this strain might have a slight delay in growth that is more evident at 24h than at 48h. The strain CPL2H1 used as parental strain in the generation of deletion mutants is auxotrophic for histidine and leucine, and consequently cannot growth on SD. Similarly, both *Ed kis1* and *Ed rha1* lack a functional copy of *MET10* and are therefore cysteine/methionine auxotrophs[15]; this phenotype is not related to the mutations introduced into *KIS1* and *RHA1*.

**Supplementary Table 1. *C. parapsilosis* CLIB214 genes involved in different cellular processes based on phenotypic screening.**

| | |
|---|---|
| **Response to antifungal drugs** | |
| Caspofungin | *PRK1, CLA4, RFX1,* |
| Fluconazole | *VPS34, 100630, GZF3, UPC2, ZCF29, ZCF31, PRK1, ISW2, ZCF38, RTG3, APD1* |
| Ketoconazole | *VPS34, SWI4, MAC1, 100660*[a]*, 802280, UPC2, ZCF1, SLN1* |
| **Response to heavy metals** | |
| Copper | *VPS34, PRK1, TUP1, TPK2, MNL1, PHO23, RTG3, CUP2, SSN3* |
| Cadmium | *VPS34, YAF9, CUP9, MAC1, TUP1, CAP1, MKK2, PBS2, PHO23, RFX1,* |
| EDTA | *MAC1, CSR1* |
| **Response to osmotic (Os), oxidative (Ox), cell wall (CW) stress** | |
| NaCl (Os) | *100910, KIS1, RNY11, PBS2, 100920, SAK1, APD1* |
| LiCl (Os) | *SAH1, PBS2, PHO23,* |
| Sorbitol (Os) | *PBS2, 100920* |
| $H_2O_2$ (Ox) | *VPS34, SWI4, MAC1, SKN7, CAP1, PBS2,* |
| Caffeine (CW) | *VPS34, RAD53* |
| **Use of alternative nitrogen sources** | |
| Alanine | *VPS15, RBF1* |
| Allantoin | *VPS15, PPR1, SAH1, RBF1* |
| Arginine | *SEF1, VPS15, CAR1, 212760, BNA2, RBF1, HAC1,* |
| Cysteine | *VPS34, SFP1, TUP1, SAH1, PFK1, PHO23, GRR1, SAK1, SSK2, SSN3, RAD53* |
| GABA | *UGA3, VPS15, SAH1, BNA2, RBF1, HAC1* |
| Glutamate | *VPS15, FGR15, SAH1, RBF1, HAC1* |
| Glutamine | *VPS15, SAH1, RBF1, HAC1* |
| Glycine | *VPS15, RBF1* |
| Isoleucine | *ARO80, VPS34* |
| Histidine | *SKN7, RFX1, VPS34, SSN3, RAD53* |
| L-ornithine | *CLA4, GZF3, SFP1, PRK1, PUT3, VPS34* |
| Tryptophan | *GAT1, ARO80, CZF1, SAH1,* |
| Leucine | *GAT1, ARO80, VPS15, PHO23, VPS34* |
| Lysine | *VPS34, PRK1, SAH1, TPK2, GRR1, BNA2, RFX1, STP4, RTG3, RFX2, SUC1, 105230, SSK2, SSN3, RAD53* |
| Methionine | *GAT1, GCN4,* |
| Proline | *ARG83, FGR15, PUT3, BNA2,* |
| Serine | *BNA2* |
| Threonine | *GAT1, ARO80, GCN4, CUP2, GCN2* |
| Valine | *ARO80, GCN4, FGR15,* |
| Urea | *NTO1, FGR3, SAH1, SSK2* |
| Aspartate | *GRR1, RTG3, VPS34* |
| **Amino acid biosynthesis** | |
| Histidine | *GCN4, GLN3* |
| Trp/Phe/Tyr | *BNA2, RAD53* |
| Asn/Thr/Met/Cys/Asp | *SAH1, BNA2, CUP2* |
| Met/Cys/Ser/Gly | *SFP1, TUP1, SAK1, RFX1, RAD53* |
| Leu/Val/Ile | *SFP1, RAD53* |
| Ala | *BNA2, RAD53* |
| Lys/Arg/Pro/Gln/Glu | *GCN4, ACE2, CUP2, RFX2, RAD53* |
| Uracil | *BNA2, SSK2, RAD53* |
| Adenine | *SFP1, SSK2, RAD53* |

See also Supplementary Data 2-3, Supplementary Figures 3-4.

[a.] *C. parapsilosis* genes lacking the functional name are indicated with the six digits ID that follows the "*CPAR2_*" prefix: for example, *100630* stands for *CPAR2_100630*

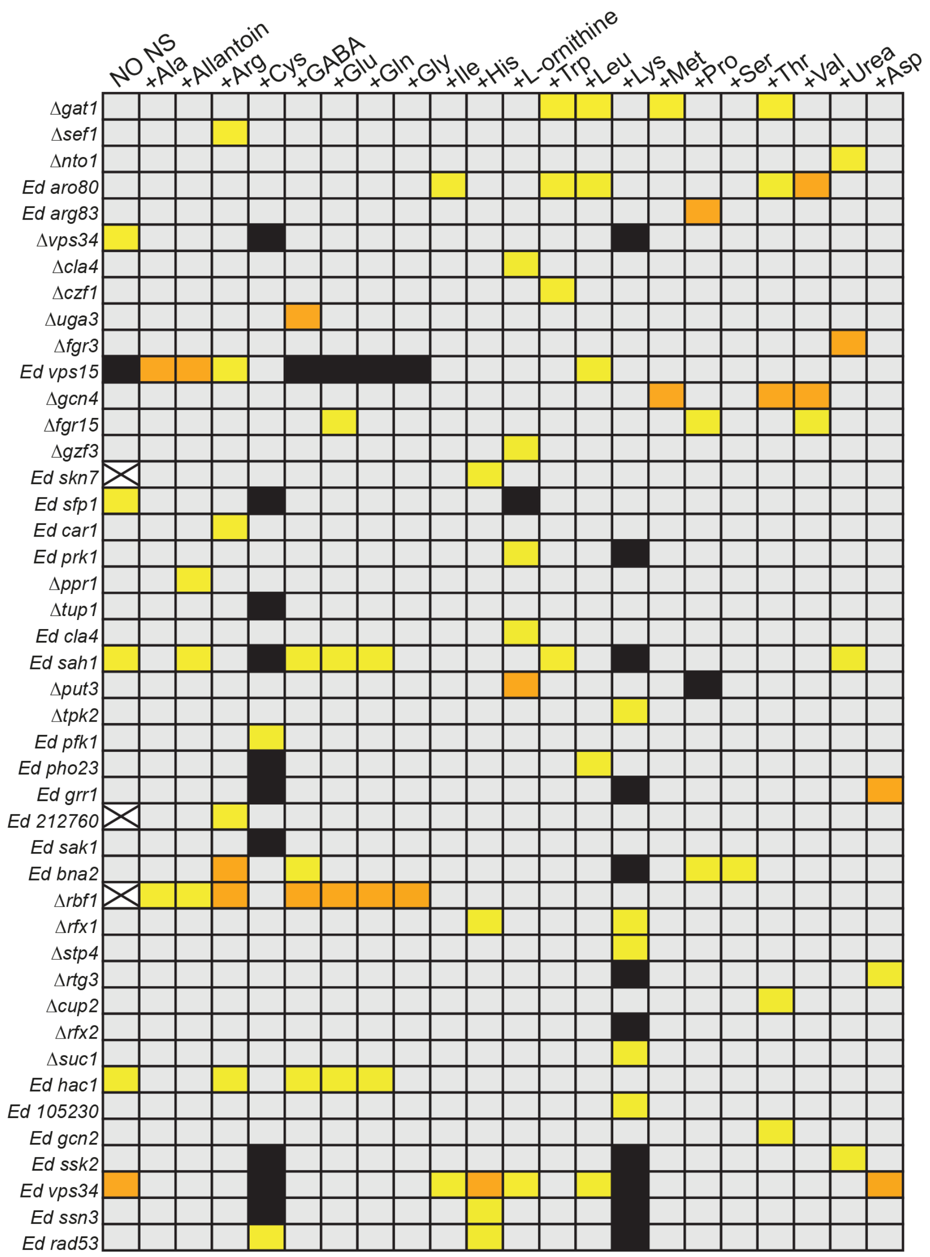


**Supplementary Figure 3. Utilization of alternative nitrogen sources by the *C. parapsilosis* mutant collection.** The ability of the mutant collection to utilize different nitrogen sources was tested (Supplementary Data 3). In order to detect growth defects compared to the control condition (YNB + ammonium sulphate), Z scores values were calculated (see Methods) and depicted in the heat-map as described in Fig 1. White crossed boxes indicate a pinning mistake.

# Supplementary Note 2

## Phenotypic effects of disruptions on biosynthesis of amino acids

We evaluated the ability of the mutant collection to synthesize L-amino acids used in protein synthesis by measuring growth in the absence of specific amino acids (Supplementary Figure 4, Supplementary Data 3). The general nitrogen regulator Gcn4 is required for synthesis of several amino acids, including histidine and glutamate, whereas deletion of *GLN3* completely abolished growth in the absence of histidine (Supplementary Figure 4, Supplementary Data 3) [14,16]. Disruption of *SFP1* (*CPAR2_807380*) affected biosynthesis of adenine, leucine, valine, isoleucine, and amino acids from the serine family (Supplementary Figure 4). In *C. albicans*, Sfp1 is a regulator of biofilm formation[17] and stress response[18,19], and the *S. cerevisiae* homolog has a role in nitrogen metabolism[20]. Deleting the transcription factors *RFX1* (*CPAR2_204990*) or *RFX2* (*CPAR2_401290*) resulted in a growth defect in the absence of the serine and glutamate amino acid families, respectively (Supplementary Figure 4). In *C. albicans* Rfx2 is involved in DNA damage response, morphogenesis, and virulence[21]. In addition, deleting *CUP2* (*CPAR2_201510*) – a transcription factor that in *C. albicans* is required for resistance to copper[22] – reduced growth when amino acids from the aspartate and glutamate families were not provided (Supplementary Figure 4).

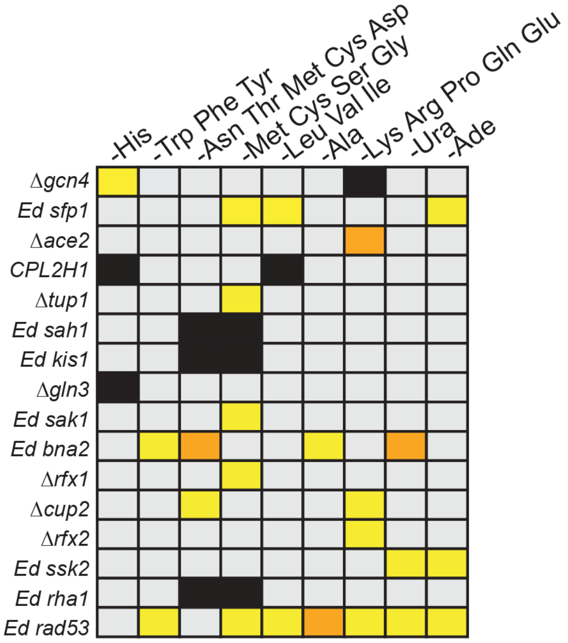


**Supplementary Figure 4. Genes involved in amino acids biosynthesis in *C. parapsilosis*.** The mutant library was replicated on SC plates lacking amino acids, uracil or adenine (Supplementary Data 3). Plates were photographed, and Z score values were calculated to determine growth defects compared to the control condition (SC supplemented with amino acids). Only mutant strains that showed a defect in at least one condition are included in the heatmap. The strain CPL2H1 used as parental strain in the generation of deletion mutants is auxotrophic for histidine and leucine, and consequently cannot growth in their absence. Both *Ed kis1* and *Ed rha1* lack a functional copy of *MET10* and are therefore cysteine/methionine auxotrophs[15]; this phenotype is not related to the mutations introduced into *KIS1* and *RHA1*. The color-coding is the same as in Fig 1.

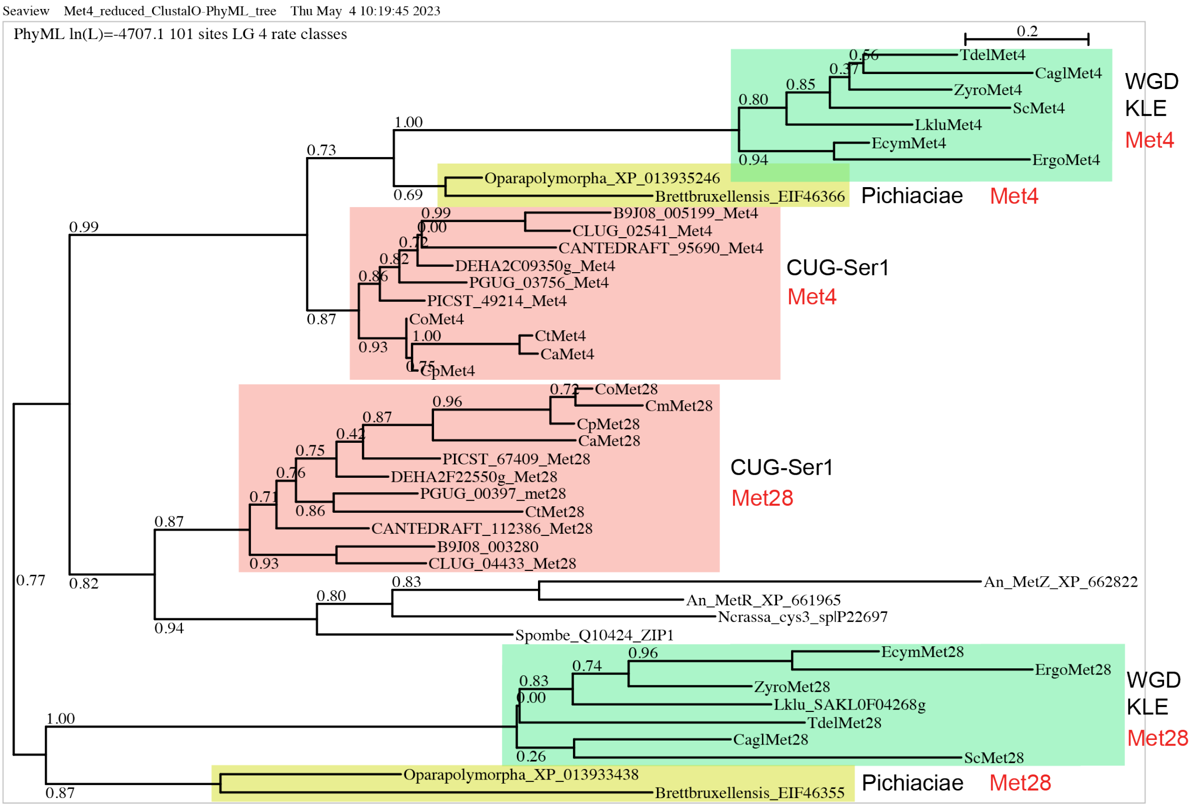


**Supplementary Figure 5. Phylogeny of the Met4 and Met28 paralogs in budding yeasts.** Protein sequences retrieved from YGOB[23], CGOB[24] or the indicated accession numbers were aligned using Muscle (as opposed to ClustalO) implemented in Seaview[25]. Trees were inferred using PhyML restricted to conserved regions selected using Gblocks. WGD = Whole Genome Duplication; KLE = *Kluyveromyces/Lachancea/Eremothecium*.

# Supplementary Note 3

**Volcano plots as described in Fig. 4*.*** We report here in the Supplementary the same plots presented in Fig 4, but in a size that allows the labelling of sulphur-related genes. The genes included in the rectangular label have characterized orthologs in *S. cerevisiae*. The genes that do not are labelled in a smaller size and by the number of their *C. parapsilosis* ID (e.g. 702100 is gene *CPAR2_702100*).

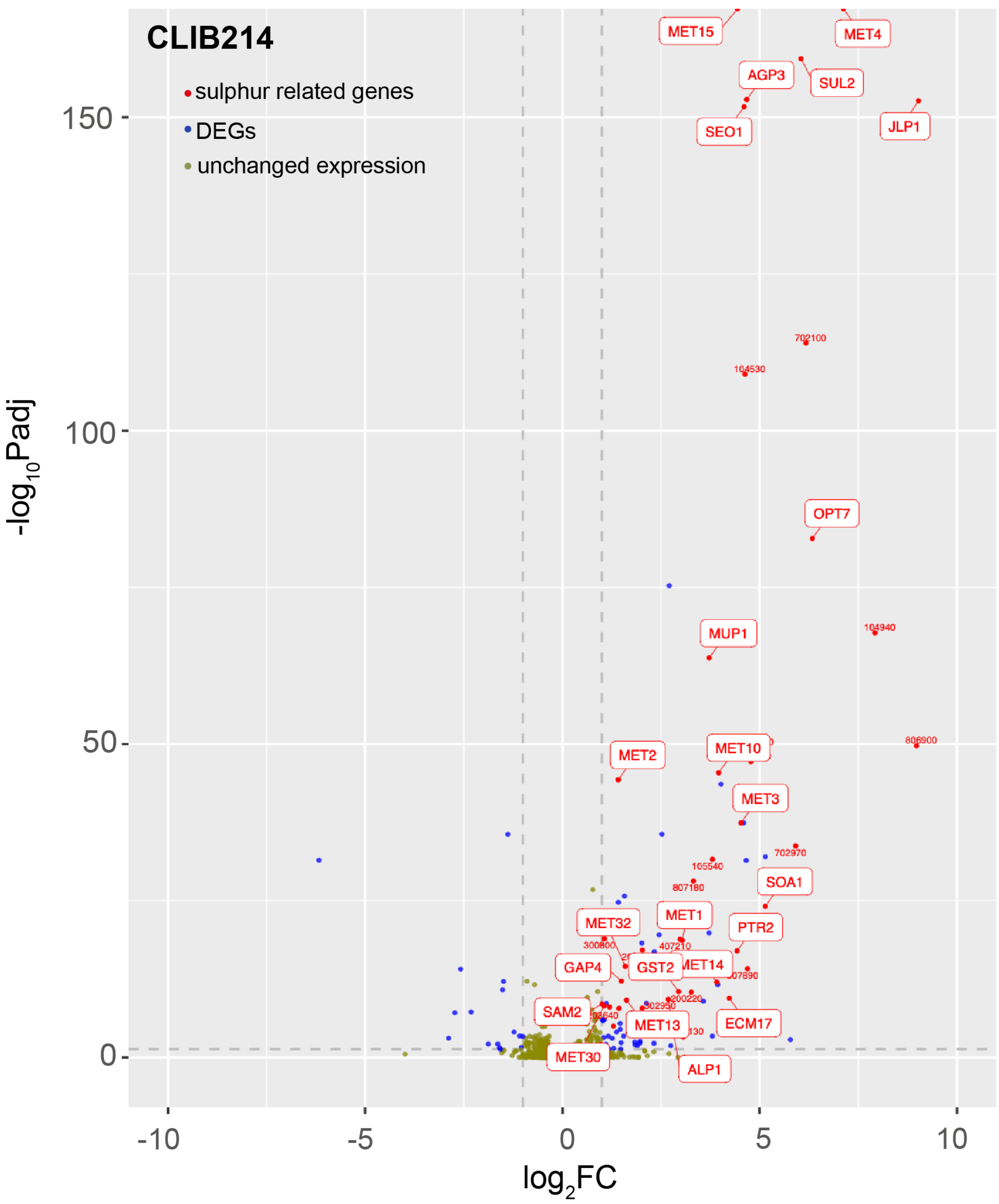


**Supplementary Figure 6. CLIB214**

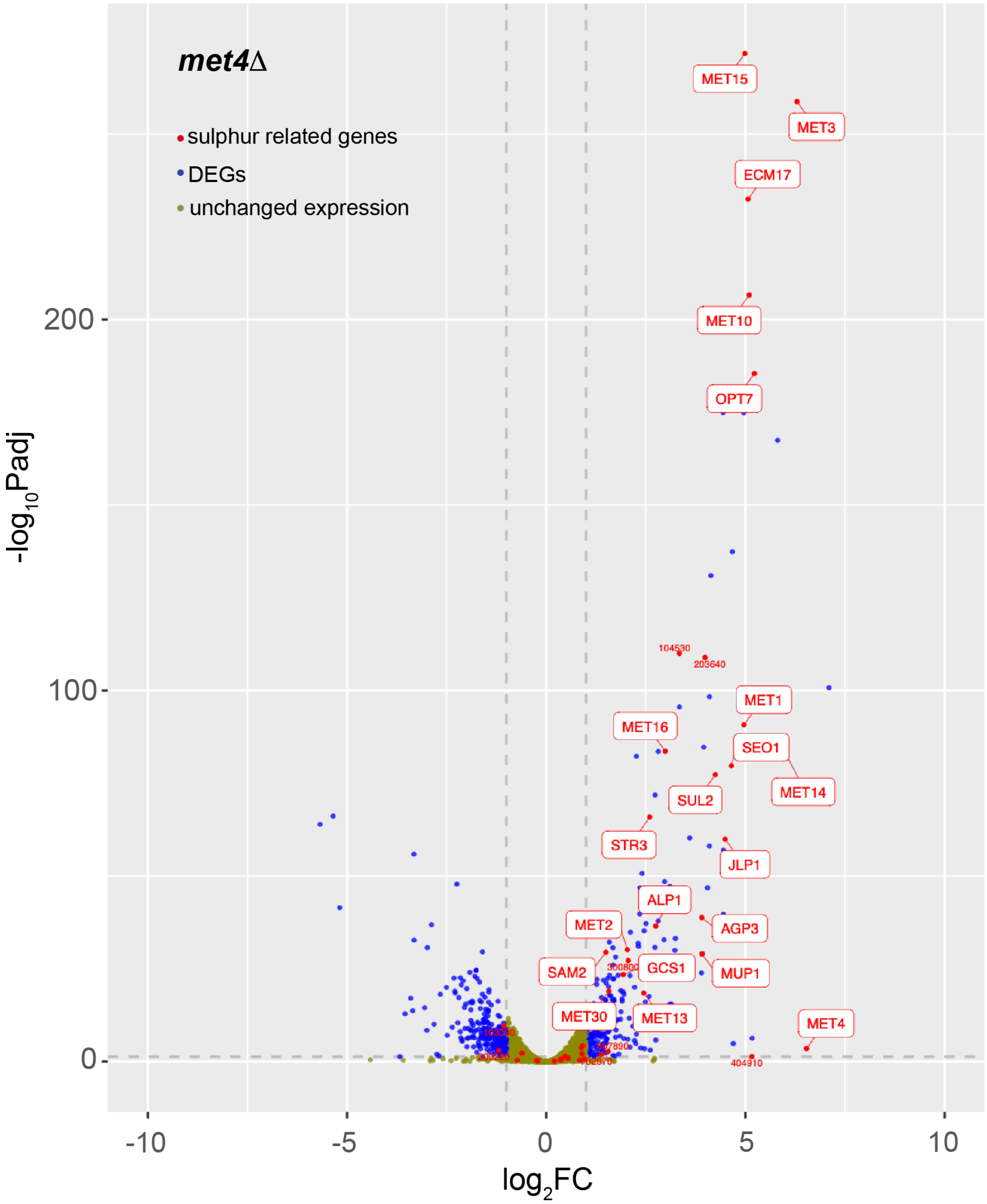


**Supplementary Figure 6. *met4Δ***

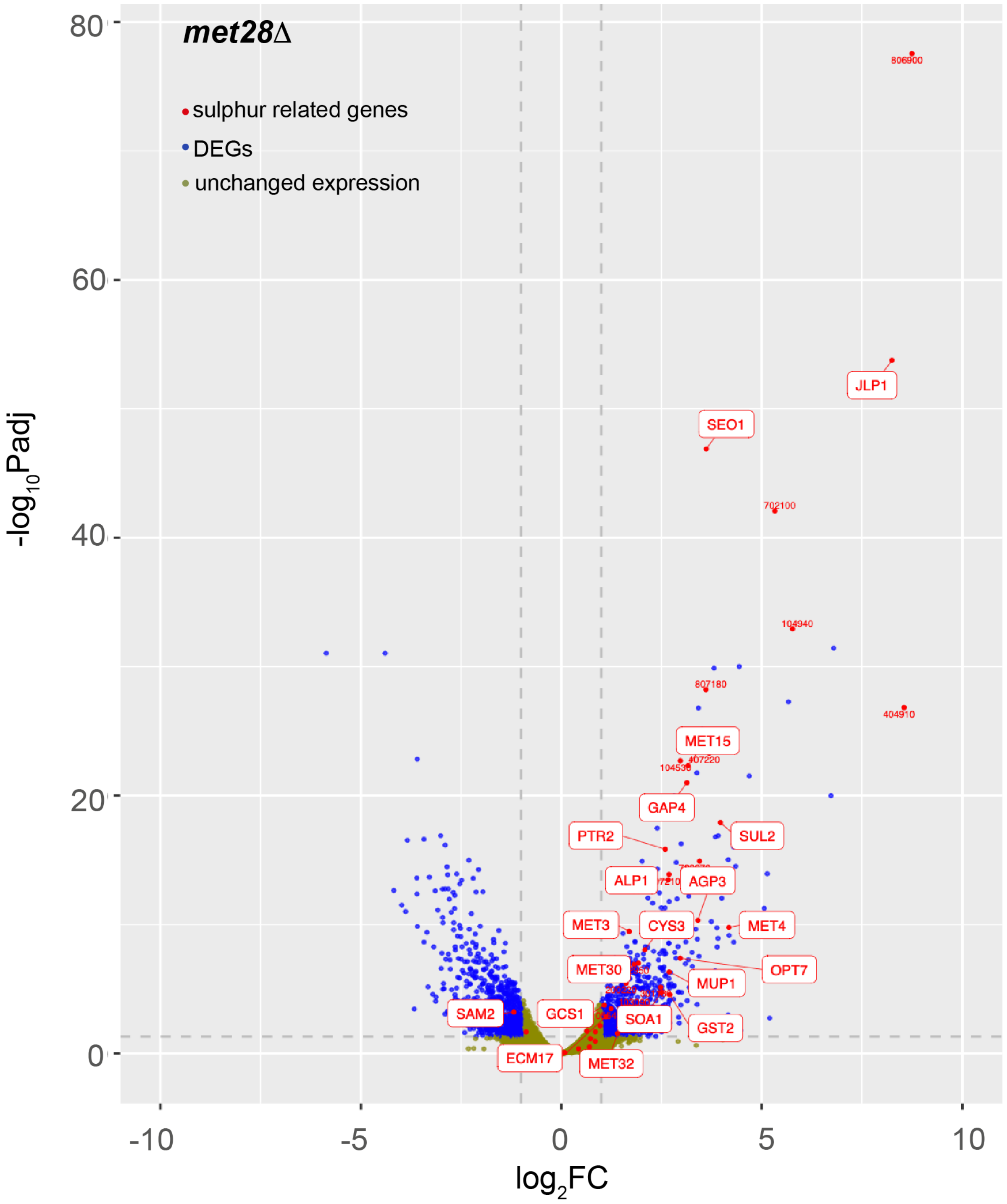


**Supplementary Figure 6.** ***met28Δ***

**Supplementary Table 2. Upregulated genes of *C. parapsilosis* CLIB214 upon Cys/Met starvation and their expression in the *met4* and *met28* single and double deletion mutants.**

| Category | CLIB214 | *met4Δ* | *met28Δ* | *me4Δ/ met28Δ* |
|---|---|---|---|---|
| TFs & regulatory proteins | *MET4* (7.1)[a], *MET32* (1.6), *MET30* (1.1), *STP1* (1) | *MET30* (1.6) | *MET4* (4.2), *MET32* (1.4), *MET30* (1.8), *STP1* (1.7) | |
| Sulphate assimilation | *MET3* (4.5), *MET15* (4.4), *ECM17* (4.2), *MET10* (4), *MET14* (3.9), *MET16* (1.9), *MET2* (1.4) | *MET3* (6.3), *MET15* (5), *ECM17* (5), *MET10* (5.1), *MET14* (5.9), *MET16* (3), *MET2* (2) | *MET3* (1.7), *MET15* (3.1), *ECM17* (1.1), | |
| Met Metabolism | *SAM2* (1), *MET1* (3), *MET13* (1.7) | *SAM2* (1.5), *MET1* (5), *MET13* (2.4) | *SAM2* (-1.2) | |
| Cys Metabolism | *CYS3* (1.3), *STR3* (1.2), *GCS1*[b] (1), *GST2* (2.9) | *STR3* (2.6), *GCS1* (2) | *CYS3* (2.1), *GCS1* (1), *GST2* (2.7) | |
| Sulphate transp. | *SUL2* (6) | *SUL2* (4.2) | *SUL2* (4) | |
| Sulphur compound transp. | *SOA1* (5.1), *SEO1* (4.6), *702970*[c] (6), *200220* (3.3), *203280* (2) | *SEO1* (4.6), *702970* (1.4) | *SOA1* (2.5), *SEO1* (3.6), *702970* (3.5), *200220* (1.6), *203280* (2.1) | |
| AA & oligopeptide transp. | *AGP3* (4.6), *MUP1* (3.7), *OPT7* (6.3), *807890* (4.7), *PTR2* (4.4), *301130* (3), *ALP1* (2.7), *GAP4* (1.5) | *AGP3* (3.9), *MUP1* (3.9), *OPT7* (5.2), *807890* (1.6), *ALP1* (2.8) | *AGP3* (3.4), *MUP1* (2.7), *OPT7* (3), *PTR2* (2.6), *301130* (2.5), *ALP1* (2.7), *GAP4* (3.1) | |
| Other transp. | *SNQ2* (1.5), *108880* (1.8), *802720* (3.8), *108340* (2.7), *YMC2* (2.7), *MDR1B* (1.8), *300590* (1.5) | *SNQ2* (1.7), *YMC2* (5), *MDR1B* (2.8) | *SNQ2* (1), *YMC2* (1). *MDR1B* (5.7), *108880* (2.5), *300590* (1.8) | *SNQ2* (1.2), *MDR1B* (1.9), *300590* (1.4) |
| 2-oxoglutarate-dep. dioxygenases | *JLP1* (9), *806900* (9), *702100* (6.2), *407220* (4.8), *104530* (4.6), *404910* (4.5), | *JLP1* (4.5), *104530* (3.3), | *JLP1* (8.2), *806900* (8.7), *702100* (5.3), *407220* (3.7), *104530* (3), *404910* (8.6) | |
| $FMNH_2$-dep. monooxygenases | *104940* (8), *807180* (3.3), *407210* (3), *302950* (2) | | *104940* (5.8), *807180* (3.6), *407210* (2.7), *302950* (1.9) | |
| Arylsulfatases | *103090* (4.8), *105540* (3.8) | | *103090* (1.7), *105540* (3.9) | |
| Oxidative stress response | *FTR1* (1.1), *ROX1* (1), *PRX1* (4.7), *210110* (5.8), *405440* (4), *601950* (2.5), *FET3* (1.7) | *ROX1* (1.5), *PRX1* (4.7), *601950* (4.1), *FET3* (-2.7) | *FTR1* (3.2), *ROX1* (2.3), *PRX1* (1.1), *405440* (3.5), *FET3* (2.2) | |
| Sugar transp. | *HGT8* (2.3), *HXT5* (1.8) | | | |
| OAS pathway | *203640* (1.4), *300800* (1.1) | *203640* (4), *300800* (2.1) | | |
| Sporulation related genes | *SMA2* (5.1), *SPO11* (4.6), *SPO75* (1.1) | *SMA2* (4.4), *SPO11* (7.1), *SPO75* (4) | *SMA2* (2.9) | |
| SAP/adhesin-like | *302640* (2.3), *805340* (2), *801790* (2) | *805340* (4.1), *801790* (2.4) | | |
| others | *DUR4* (4), *807040* (3.7), *ARO9* (2.1), *TPO3* (1.3), *FDH1* (1.8), *BNA3* (1.4), *503870* (1.6), *500140* (1.4), *PGA23* (1.3), *CHA1* (1.2), *212190* (1.1) | *807040* (5.2), *TPO3* (2.2), *BNA3* (1.4), *503870* (2.3), *500140* (6), *PGA23* (2.6), *CHA1* (2), | *ARO9* (1.4), *DUR4* (4.7), *TPO3* (3.4), *CHA1* (3.4), *212190* (1.6) | *CHA1* (1.8), |
| Unknown function | *210700* (3.6), *102480* (2.5), *101750* (2.1), *203580* (2), *201160* (2), *704060* (1.9), *809010* (1.7), *502110* (1.6), *210410* (1.5), *203090* (1.5), *103320* (1.2), *400960* (1.1), *703250* (1.1), *805910* (1) | *203580* (1.7), *201160* (1.8), *809010* (2.5), *203090* (2.7) | *210700* (2.4), *101750* (1.5), *203580* (2.5), *201160* (3.4), *210410* (1.2), *203090* (3.4), *103320* (1.7), *703250* (2.2), *805910* (1.8) | *703250* (1.3) |

Genes related to sulphur are highlighted in red in CLIB214. For the complete list of differentially regulated genes and the exact P values: Supplementary Data 4. The Wald Test was used as implemented in the DESeq2 package for statistical analysis. An implementation of the Benjamini-Hochberg false discovery rate (FDR) multiple test correction was applied in DESeq2. The genes *CPAR2_402890*, *CPAR2_404910*, and *CPAR2_402070* were not included in the *met4Δ* column because one of the conditions in the data set had zero reads (Supplementary Data 4).

[a.] numbers in brackets indicate the $Log_2FC$ in expression (SC -Cys/Met versus SC + AA) versus; only genes with adjusted p-value <0.05 are shown.

[b.] *GCS1* had a logFC = 0.98, so it was included.

[c.] *C. parapsilosis* genes lacking the functional name are indicated with the six digits ID that follows the "*CPAR2_*" prefix: for example, *702970* stands for *CPAR2_702970*

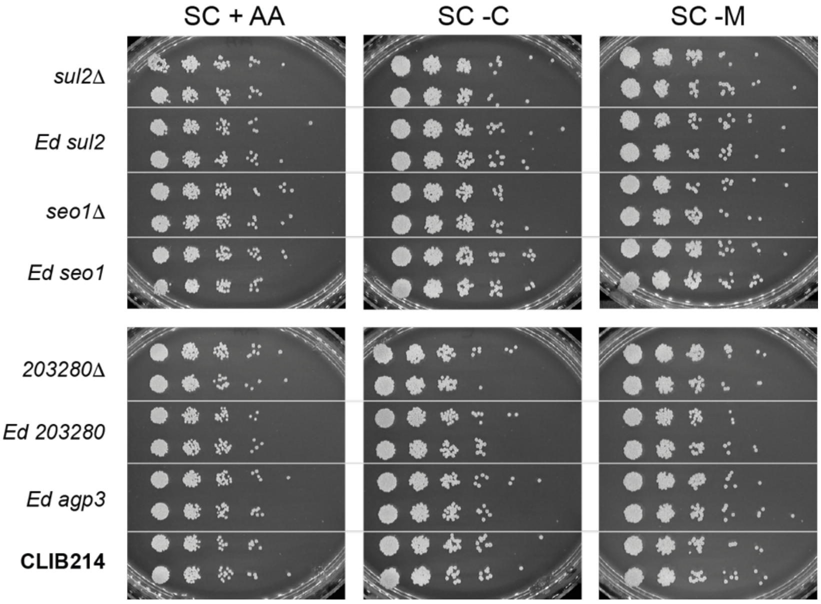


**Supplementary Figure 7. Loss of genes regulated by both Met4 and Met28 does not result in Met auxotrophy.** Three genes whose transcription is regulated equally by Met4 and Met28 (see Fig 5) were disrupted by insertion of a premature stop codon or deleted (*SUL2, SEO1, AGP3*). Although *CPAR2_203280* is regulated by Met4 (Fig 5), it was included because this gene and *SEO1* are 88% identical. All strains could grow in the absence of Cys or Met. *C. parapsilosis* CLIB214 was included as control. Source data are provided as a Source Data file.

**Supplementary Table 3. Binding motifs recognized by Met32 and Cbf1 in the promoters of sulphur-related genes induced under Cys/Met starvation.**

| Gene | Met32 motifs | Cbf1 motifs |
|---|---|---|
| *CPAR2_200220* | -198, -447, -650, -839 | |
| *CPAR2_203280* | -883 | -795 |
| *SOA1* | +571, -626, -683 | |
| *MET32* | +160, +271, -373, -871 | |
| *CPAR2_806900* | -546, -706, +819 | |
| *CPAR2_702100* | +456, +731, -859 | |
| *CPAR2_407220* | -817, -881 | |
| *CPAR2_404910* | +91, +579, +656, +774, +933, -969 | |
| *CPAR2_104940* | -458, +820 | +191 |
| *CPAR2_807180* | +297, -383, +864 | |
| *CPAR2_407210* | +891 | |
| *CPAR2_302950* | -795, -896 | |
| *CPAR2_103090* | +881 | |
| *CPAR2_105540* | +857 | |
| *CYS3* | -218, +266, -727, -832 | -51, +702 |
| *GST2* | -875 | |
| *PTR2* | -245, -856 | |
| *CPAR2_301130* | +331, -462, -515, | |
| *GAP4* | +751, -933 | |
| *MET14* | | +850 |
| *MET16* | | |
| *MET10* | | -174, +190, -862 |
| *MET2* | -804 | +581 |
| *MET13* | | -563 |
| *SAM2* | -90, +761 | |
| *MET1* | | |
| *STR3* | | -778 |
| *CPAR2_203640* | | |
| *CPAR2_300800* | | +852 |
| *CPAR2_807890* | -223, -614, -896 | |
| *SUL2* | -143, -582, | |
| *SEO1* | -721, -866 | +538 |
| *CPAR2_702970* | -655, -748 | |
| *AGP3* | | |
| *MET30* | +148, -280, -617, -960 | +358, +936 |
| *MUP1* | -149, +545, -748, | -326, |
| *JLP1* | +461, +731 | |
| *CPAR2_104530* | +902 | |
| *MET3* | -480 | +795 |
| *ECM17* | | +188, -677 |
| *MET15* | -829 | +762 |
| *GCS1* | -899 | +177 |
| *OPT7* | +509, +616 | +796 |
| *ALP1* | +809 | -343 |
| *MET4* | +274, +670, -828 | +531 |

1 Kb upstream of the ATG was considered. Position 0 = - 1000 bp upstream of ATG; position 1000 = - 1 bp upstream of ATG. "+" = coding strand; "-" = reverse strand. The sites were detected using the MEME-suite (MEME and MAST) [26].

## Supplementary References


1 Holland, L. M. *et al.* Comparative phenotypic analysis of the major fungal pathogens *Candida parapsilosis* and *Candida albicans*. *PLoS Pathog* **10**, e1004365 (2014). https://doi.org:10.1371/journal.ppat.1004365

2 Lombardi, L., Oliveira-Pacheco, J. & Butler, G. Plasmid-based CRISPR-Cas9 gene editing in multiple *Candida* Species. *mSphere* **4** (2019). https://doi.org:10.1128/msphere.00125-19

3 Sellam, A. *et al.* The p38/HOG stress-activated protein kinase network couples growth to division in *Candida albicans*. *PLoS Genet* **15**, e1008052 (2019). https://doi.org:10.1371/journal.pgen.1008052

4 Noble, S. M., French, S., Kohn, L. A., Chen, V. & Johnson, A. D. Systematic screens of a *Candida albicans* homozygous deletion library decouple morphogenetic switching and pathogenicity. *Nat Genet* **42**, 590-598 (2010). https://doi.org:10.1038/ng.605

5 Jorgensen, P., Nishikawa, J. L., Breitkreutz, B. J. & Tyers, M. Systematic identification of pathways that couple cell growth and division in yeast. *Science* **297**, 395-400 (2002). https://doi.org:10.1126/science.1070850

6 Oh, J. *et al.* Gene annotation and drug target discovery in *Candida albicans* with a tagged transposon mutant collection. *PLoS Pathog* **6**, e1001140 (2010). https://doi.org:10.1371/journal.ppat.1001140

7 Ramírez-Zavala, B. *et al.* The Snf1-activating kinase Sak1 is a key regulator of metabolic adaptation and *in vivo* fitness of *Candida albicans*. *Mol Microbiol* **104**, 989-1007 (2017). https://doi.org:10.1111/mmi.13674

8 Yoshikawa, K. *et al.* Comprehensive phenotypic analysis of single-gene deletion and overexpression strains of *Saccharomyces cerevisiae*. *Yeast* **28**, 349-361 (2011). https://doi.org:10.1002/yea.1843

9 Segal, E. S. *et al.* Gene Essentiality Analyzed by In Vivo Transposon Mutagenesis and Machine Learning in a Stable Haploid Isolate of *Candida albicans*. *mBio* **9** (2018). https://doi.org:10.1128/mBio.02048-18

10 Giaever, G. *et al.* Functional profiling of the *Saccharomyces cerevisiae* genome. *Nature* **418**, 387-391 (2002). https://doi.org:10.1038/nature00935

11 Bruckmann, A., Künkel, W., Härtl, A., Wetzker, R. & Eck, R. A phosphatidylinositol 3-kinase of *Candida albicans* influences adhesion, filamentous growth and virulence. *Microbiology (Reading)* **146 ( Pt 11)**, 2755-2764 (2000). https://doi.org:10.1099/00221287-146-11-2755

12 Kitanovic, A. *et al.* Phosphatidylinositol 3-kinase VPS34 of *Candida albicans* is involved in filamentous growth, secretion of aspartic proteases, and intracellular detoxification. *FEMS Yeast Res* **5**, 431-439 (2005). https://doi.org:10.1016/j.femsyr.2004.11.005

13 Blankenship, J. R., Fanning, S., Hamaker, J. J. & Mitchell, A. P. An extensive circuitry for cell wall regulation in *Candida albicans*. *PLoS Pathog* **6**, e1000752 (2010). https://doi.org:10.1371/journal.ppat.1000752

14 Turner, S. A., Ma, Q., Ola, M., Martinez de San Vicente, K. & Butler, G. Dal81 Regulates Expression of Arginine Metabolism Genes in *Candida parapsilosis*. *mSphere* **3** (2018). https://doi.org:10.1128/mSphere.00028-18

15 Lombardi, L., Bergin, S. A., Ryan, A., Zuniga-Soto, E. & Butler, G. CRISPR-Cas9 editing induces Loss of Heterozygosity in the pathogenic yeast *Candida parapsilosis*. *mSphere* **7**, e00393-00322 (2022). https://doi.org:doi:10.1128/msphere.00393-22

16 Ljungdahl, P. O. & Daignan-Fornier, B. Regulation of amino acid, nucleotide, and phosphate metabolism in *Saccharomyces cerevisiae*. *Genetics* **190**, 885-929 (2012). https://doi.org:10.1534/genetics.111.133306

17 Chen, H. F. & Lan, C. Y. Role of *SFP1* in the Regulation of *Candida albicans* Biofilm Formation. *PLoS One* **10**, e0129903 (2015). https://doi.org:10.1371/journal.pone.0129903

18 Lee, S. Y., Chen, H. F., Yeh, Y. C., Xue, Y. P. & Lan, C. Y. The Transcription Factor Sfp1 Regulates the Oxidative Stress Response in *Candida albicans*. *Microorganisms* **7** (2019). https://doi.org:10.3390/microorganisms7050131

19 Kastora, S. L., Herrero-de-Dios, C., Avelar, G. M., Munro, C. A. & Brown, A. J. P. Sfp1 and Rtg3 reciprocally modulate carbon source-conditional stress adaptation in the pathogenic yeast *Candida albicans*. *Mol Microbiol* **105**, 620-636 (2017). https://doi.org:10.1111/mmi.13722

20 VanderSluis, B. *et al.* Broad metabolic sensitivity profiling of a prototrophic yeast deletion collection. *Genome Biol* **15**, R64 (2014). https://doi.org:10.1186/gb-2014-15-4-r64

21 Hao, B. *et al. Candida albicans RFX2* encodes a DNA binding protein involved in DNA damage responses, morphogenesis, and virulence. *Eukaryot Cell* **8**, 627-639 (2009). https://doi.org:10.1128/ec.00246-08

22 Douglas, L. M. & Konopka, J. B. Plasma membrane architecture protects *Candida albicans* from killing by copper. *PLoS Genet* **15**, e1007911 (2019). https://doi.org:10.1371/journal.pgen.1007911

23 Byrne, K. P. & Wolfe, K. H. The Yeast Gene Order Browser: combining curated homology and syntenic context reveals gene fate in polyploid species. *Genome Res* **15**, 1456-1461 (2005). https://doi.org:10.1101/gr.3672305

24 Maguire, S. L. *et al.* Comparative genome analysis and gene finding in *Candida* species using CGOB. *Mol Biol Evol* **30**, 1281-1291 (2013). https://doi.org:10.1093/molbev/mst042

25 Gouy, M., Guindon, S. & Gascuel, O. SeaView version 4: A multiplatform graphical user interface for sequence alignment and phylogenetic tree building. *Mol Biol Evol* **27**, 221-224 (2010). https://doi.org:10.1093/molbev/msp259

26 Bailey, T. L., Johnson, J., Grant, C. E. & Noble, W. S. The MEME Suite. *Nucleic Acids Research* **43**, W39-W49 (2015). https://doi.org:10.1093/nar/gkv416
