# Supplementary material for: Alternative sulphur metabolism in the fungal pathogen Candida parapsilosis
Source: Nat Commun. 2024 Oct 24;15:9190. doi: 10.1038/s41467-024-53442-8 (PMC11502921; doi:10.1038/s41467-024-53442-8)
Supplement: Supplementary file 2 — Description of Additional Supplementary Files [file 41467_2024_53442_MOESM2_ESM.pdf]

Description of Additional Supplementary Files:

Supplementary Data 1: List of strains and oligonucleotides used in the study

Supplementary Data 2: Growth on the library on SC, YNB and YPD media

Supplementary Data 3: Growth of the library in phenotyping conditions

Supplementary Data 4: Differential expression analysis
