# Supplementary material for: Alternative sulphur metabolism in the fungal pathogen Candida parapsilosis
Source: Nat Commun. 2024 Oct 24;15:9190. doi: 10.1038/s41467-024-53442-8 (PMC11502921; doi:10.1038/s41467-024-53442-8)
Supplement: Supplementary file 7 — Reporting Summary [file 41467_2024_53442_MOESM7_ESM.pdf]

nature portfolio

Corresponding author(s): Lisa Lombardi, Geraldine Butler

Last updated by author(s): 23/09/2024

Reporting Summary

Nature Portfolio wishes to improve the reproducibility of the work that we publish. This form provides structure for consistency and transparency in reporting. For further information on Nature Portfolio policies, see our Editorial Policies and the Editorial Policy Checklist.

Statistics

For all statistical analyses, confirm that the following items are present in the figure legend, table legend, main text, or Methods section.

n/a	Confirmed	
☐	☒	The exact sample size (*n*) for each experimental group/condition, given as a discrete number and unit of measurement
☐	☒	A statement on whether measurements were taken from distinct samples or whether the same sample was measured repeatedly
☐	☒	The statistical test(s) used AND whether they are one- or two-sided<br>*Only common tests should be described solely by name; describe more complex techniques in the Methods section.*
☐	☒	A description of all covariates tested
☐	☒	A description of any assumptions or corrections, such as tests of normality and adjustment for multiple comparisons
☐	☒	A full description of the statistical parameters including central tendency (e.g. means) or other basic estimates (e.g. regression coefficient) AND variation (e.g. standard deviation) or associated estimates of uncertainty (e.g. confidence intervals)
☐	☒	For null hypothesis testing, the test statistic (e.g. *F*, *t*, *r*) with confidence intervals, effect sizes, degrees of freedom and *P* value noted<br>*Give P values as exact values whenever suitable.*
☒	☐	For Bayesian analysis, information on the choice of priors and Markov chain Monte Carlo settings
☒	☐	For hierarchical and complex designs, identification of the appropriate level for tests and full reporting of outcomes
☒	☐	Estimates of effect sizes (e.g. Cohen's *d*, Pearson's *r*), indicating how they were calculated

Our web collection on statistics for biologists contains articles on many of the points above.

Software and code

Policy information about availability of computer code

Data collection	Software used for the data collection are described in the Methods section. Commercially available software used included Microsoft Excel. Growth on phenotyping plates was assessed using the open-source Phenobooth web-based application (https://singerinstruments.shinyapps.io/phenobooth/).
Data analysis	Software used for the data analysis are described in the Methods section. Commercially available software used included Microsoft Excel. For the RNA-seq analysis reads were aligned to the genome using STAR (v. 2.7.3a). HTSeq (v. 0.13.5) was used to count mapped reads per gene. Differentially expressed genes were identified using DESeq2 in R (v.3.5.0). From the MEME-suite (v. 5.4.0) the MEME and MAST programs were used to identify motifs upstream of the sulphur related genes induced in sulphur starvation. For statistical analysis the open-source web-based application StatsKingdom (https://www.statskingdom.com) was used.

For manuscripts utilizing custom algorithms or software that are central to the research but not yet described in published literature, software must be made available to editors and reviewers. We strongly encourage code deposition in a community repository (e.g. GitHub). See the Nature Portfolio guidelines for submitting code & software for further information.

Data

Policy information about availability of data

All manuscripts must include a data availability statement. This statement should provide the following information, where applicable:

- Accession codes, unique identifiers, or web links for publicly available datasets
- A description of any restrictions on data availability
- For clinical datasets or third party data, please ensure that the statement adheres to our policy

All data generated or analysed during this study are included in this published article (and its supplementary information files). All RNA-seq data is publicly available from SRA with BioProject accession number PRJNA1044317.

Research involving human participants, their data, or biological material

Policy information about studies with human participants or human data. See also policy information about sex, gender (identity/presentation), and sexual orientation and race, ethnicity and racism.

Reporting on sex and gender	N.A.
Reporting on race, ethnicity, or other socially relevant groupings	N.A.
Population characteristics	N.A.
Recruitment	N.A.
Ethics oversight	N.A.

Note that full information on the approval of the study protocol must also be provided in the manuscript.

Field-specific reporting

Please select the one below that is the best fit for your research. If you are not sure, read the appropriate sections before making your selection.

☒ Life sciences ☐ Behavioural & social sciences ☐ Ecological, evolutionary & environmental sciences

For a reference copy of the document with all sections, see nature.com/documents/nr-reporting-summary-flat.pdf

Life sciences study design

All studies must disclose on these points even when the disclosure is negative.

Sample size	No statistical tests were utilized to predetermine sample size. Multiple samples were used for RNA-seq analysis (Fig. 4), for growth profiling in sulphur sources (Fig 7) and for biofilm assays (Fig 8). For the RNA-seq analysis, 3 replicates were used, which is the standard in the field. For the growth profiling in sulphur sources (Fig 7), at least three replicates were measured for each strain examined. For biofilm analysis, at least 3 replicates were measured, which again is the standard used. The only exception is the measurement of strain Ed met4/met28 in the absence of methionine (Figure 8). This strain is auxotrophic and cannot grow in the absence of methionine. It therefore cannot make biofilm, the measurement is zero, and there is no variation around zero. The assay was completed twice to ensure that the value is zero.
Data exclusions	No data points were excluded from the analyses.
Replication	To verify the reproducibility of the experimental findings, all experiments included at least three biological replicates (or two for one assay with a zero measurement as described above). All replication attempts were successful.
Randomization	Randomization is not relevant in our experimental setting, as we are dealing with direct comparisons between different strains.
Blinding	Blinding was not required because strains were directly compared.

Reporting for specific materials, systems and methods

We require information from authors about some types of materials, experimental systems and methods used in many studies. Here, indicate whether each material, system or method listed is relevant to your study. If you are not sure if a list item applies to your research, read the appropriate section before selecting a response.

Materials & experimental systems

n/a	Involved in the study
☒	☐ Antibodies
☒	☐ Eukaryotic cell lines
☒	☐ Palaeontology and archaeology
☒	☐ Animals and other organisms
☒	☐ Clinical data
☒	☐ Dual use research of concern
☒	☐ Plants

Methods

n/a	Involved in the study
☒	☐ ChIP-seq
☒	☐ Flow cytometry
☒	☐ MRI-based neuroimaging

Plants

Seed stocks	*Report on the source of all seed stocks or other plant material used. If applicable, state the seed stock centre and catalogue number. If plant specimens were collected from the field, describe the collection location, date and sampling procedures.*
Novel plant genotypes	*Describe the methods by which all novel plant genotypes were produced. This includes those generated by transgenic approaches, gene editing, chemical/radiation-based mutagenesis and hybridization. For transgenic lines, describe the transformation method, the number of independent lines analyzed and the generation upon which experiments were performed. For gene-edited lines, describe the editor used, the endogenous sequence targeted for editing, the targeting guide RNA sequence (if applicable) and how the editor was applied.*
Authentication	*Describe any authentication procedures for each seed stock used or novel genotype generated. Describe any experiments used to assess the effect of a mutation and, where applicable, how potential secondary effects (e.g. second site T-DNA insertions, mosiacism, off-target gene editing) were examined.*
